# Emotion regulation strategies among young men in rural areas and urban informal settlements in South Africa: a qualitative exploratory study

**DOI:** 10.1080/17450128.2025.2600959

**Published:** 2025-12-11

**Authors:** Princess Nyoni, Andrew Tomita, Smanga Mkhwanazi, Andrew Gibbs

**Affiliations:** a School of Nursing and Public Health, Discipline of Public Health Medicine, University of KwaZulu-Natal, Durban, South Africa; b Health Economics and HIV and AIDS Research Division (HEARD), University of KwaZulu-Natal, Durban, South Africa; c Centre for Rural Health, School of Nursing and Public Health, University of KwaZulu-Natal, Durban, South Africa; d KwaZulu-Natal Research Innovation and Sequencing Platform (KRISP), College of Health Sciences, University of KwaZulu-Natal, Durban, South Africa; e Gender and Health Research Unit, South African Medical Research Council, Pretoria, South Africa; f Department of Psychology, University of Exeter, Exeter, UK

**Keywords:** Emotional dysregulation, young men, marginalized communities, South Africa

## Abstract

Emotional dysregulation (ED), the inability to manage emotional responses effectively is a well-documented major symptom of poor mental health, especially in high-income countries. Evidence from these settings links ED to adverse childhood experiences, chronic poverty, and structural disadvantage. ED has also been associated with increased risk of HIV acquisition and intimate partner violence (IPV) perpetration, particularly among men. Despite these established associations, research on ED remains limited in sub-Saharan Africa, including South Africa, where both HIV and IPV remain epidemicespecially in low-resource communities such as rural areas and urban informal settlements. Therefore, we explored emotion regulation strategies among young men (aged 18–30) living in urban informal settlements and rural communities in KwaZulu-Natal (KZN), South Africa. We conducted 22 audio-recorded face-to-face interviews using a semi-structured interview guide, guided by the six domains of ED in Difficulties in Emotion Regulation Scale (DERS-16) as our conceptual framework. Data were analysed deductively using the domains of ED in DERS-16 as main themes. Participants seemed to struggle with emotion regulation likely resulting in ED, which was probably attributed to adverse childhood experiences, poverty and intensified by local constructions of masculinity. Common but ineffective emotion regulation strategies included emotion suppression and numbing with alcohol and sex, aligned with dominant masculine norms. Men’s struggles with impulsivity resulting from ED frequently manifested as violence perpetration, notably against female partners. ED seemed to also compromise men’s ability to achieve personal goals and establish stable livelihoods. Participants’ emotion regulation descriptions closely matched ED domains on DERS-16 suggesting its potential suitability in measuring ED among this population. Emotional dysregulation appears prevalent among men in marginalized KZN communities and is closely linked to increased risks of IPV and HIV. Future research is necessary to deepen understanding of these connections and inform effective interventions.

## Introduction

Growing evidence suggests a significant burden of poor mental health among young individuals in sub-Saharan Africa, including South Africa (Jörns-Presentati et al., 2021). Young men, particularly those in marginalised communities like rural areas and urban informal settlements, appear disproportionately affected by poor mental health. Both rural areas and urban informal settlements have limited economic activities and lack basic services such as clean running tap water (du Toit, 2019; Oldewage-Theron & Slabbert, 2010) and poverty indicators such as food insecurity, are as high as 90% (Mkhize & Sibanda, 2022). The stress resulting from men’s inability to fulfil 'traditional' expectations such as earning a livelihood and providing for their families might negatively impact their mental health. A recent study conducted in urban informal settlements in South Africa found notably high levels of depression (46.8%) and post-traumatic stress (14.4%) among men in these contexts (Oyekunle et al., 2023).

Emotional dysregulation (ED), typically considered a transdiagnostic factor in psychopathology (i.e. an underlying mechanism in various mental health conditions), has not been studied much in men in Africa including in urban informal settlements and rural areas in South Africa. ED can be defined as the inability to regulate negative emotions healthily or an emotional response to external stimuli that is poorly regulated and does not fall within the conventionally accepted range of emotional responses (Tacchini & Vismara, 2019), for instance, exaggerated anger outbursts. Some of the main dimensions of ED are non-acceptance of emotional responses, difficulty in engaging in goal-oriented behaviours, lack of confidence in or limited access to effective emotion regulation skills, difficulties controlling impulsive behaviours when distressed, and a lack of emotional awareness and clarity (Gratz & Roemer, 2004).

Structural factors such as adverse childhood experiences (Priyam & Nath, 2021) and poverty (Erhart et al., 2019) have been reported to diminish emotion regulation capabilities, likely leading to ED. Some studies have also suggested that dominant forms of masculinity among men may encourage emotion suppression (River & Flood, 2021), hindering emotional awareness and processing, causing emotions to build up and reducing one’s ability to respond adaptively, eventually leading to difficulty controlling emotional responses leading to ED (Chen et al., 2025). Studies have also suggested that poor mental health might aggravate ED, for instance, stress, an emotional state that can worsen mental health outcomes, has been reported to be associated with increased ED (Herts et al., 2012).

ED has critical implications including difficulties in interpersonal relationships (Garofalo et al., 2017), deteriorating mental health outcomes (Menefee et al., 2022) and of particular concern, violence perpetration and risky sexual behaviours, potentially contributing to intimate partner violence (IPV) and HIV which are both epidemic in South Africa. Although evidence linking ED to violence perpetration and risky sexual behaviour is established, most studies have been conducted outside Africa. For instance, research from the United States of America (U.S.A.) identified a heightened risk of IPV perpetration associated with ED in men (Conzemius et al., 2021). Another study conducted in the U.S.A., and a review focusing mainly on countries from the North, have also found that ED is associated with risky sexual behaviour (Tull et al., 2012; Weiss et al., 2015), which is a major risk factor of HIV acquisition.

Despite the potential significance of ED in understanding mental health, IPV, and HIV risks, there is limited research among young men in sub-Saharan Africa, including in South Africa, especially in contexts like rural areas and urban informal settlements which are characterized by severe poverty and economic inactivity (Bila & Biyase, 2022). In South Africa and including in KwaZulu-Natal, which has among the highest HIV prevalence globally (Human Science Research Council, 2024) and has reported high rates of IPV particularly in urban informal settlements (Gibbs et al., 2018), sociocultural expectations surrounding masculinity strongly influence how young men experience, interpret, and express emotional distress (Gibbs et al., 2022). Emotional expression among men in KwaZulu-Natal is shaped by long-standing cultural norms emphasising strength, composure, and social responsibility for instance IsiZulu cultural principles such as, *ukuzimela* (self-reliance), and *ukuzithiba* (self-control and restraint) valorise emotional endurance and discourage overt displays of vulnerability (Moonsamy & Thavanesi, 2024). These expectations intersect with structural pressures such as unemployment, financial strain, and social insecurity to produce environments in which emotional suppression becomes a socially sanctioned coping response. Such norms shape how emotions are recognised, regulated, and communicated, and may contribute to the adoption of suppression-based strategies that over time heighten vulnerability to ED.

A common scale to measure ED is the short version of the Difficulties in Emotion Regulation Scale (DERS-16) (Bjureberg et al., 2016), which consists of 16 items that assess how an individual regulates emotions when experiencing negative feelings (specifically being upset). The items of the DERS-16 are guided by six main domains of ED: non-acceptance of negative emotions, difficulty in engaging in goal-oriented behaviours when distressed, limited access to effective emotion regulation skills, difficulties in controlling impulsive behaviours when distressed, lack of emotional awareness, and lack of emotional clarity. Although not widely utilized in South Africa, the DERS-16 is validated internationally as reliable (Demirpence Secinti & Sen, 2023; Fekih-Romdhane et al., 2023; Ward-Smith et al., 2024) with one study confirming its superior psychometric properties compared to other short versions (Burton et al., 2022). Since the DERS-16 was developed in Western settings and has been used sparingly in South African population, some of its constructs may not fully capture locally embedded emotional expressions and coping norms hence the DERS-16 domains were used as a flexible heuristic framework, while remaining attentive to culturally grounded expressions and meanings throughout analysis.

This study aimed to explore emotion regulation strategies among young men in urban informal settlements and rural areas in KwaZulu-Natal, South Africa using the main domains of ED in the DERS-16 as a framework to guide our approach.

## Methods

We conducted an exploratory qualitative study in urban informal settlements and a rural community in KwaZulu-Natal to understand the emotion regulation capabilities of men in this context by conducting in-depth interviews using a semi-structured interview guide guided by the six domains of ED in the DERS-16.

### Study setting

The study was conducted at two sites: urban informal settings and a rural setting in KwaZulu-Natal, South Africa.

Our study was undertaken in urban informal settlements in Durban, South Africa. As the largest region in KwaZulu-Natal, there were an estimated 587 informal settlements in Durban in 2022, accounting for approximately one-quarter of its population (Misselhorn, 2022). Informal settlements are defined as communities typically built on illegal land and whose plans are not approved and are usually made of materials such as cardboard and metal sheets with minimal or absent of services such as water, sanitation and electricity (Misselhorn, 2022).

The rural community is in the northeastern KwaZulu-Natal Province. Similar to urban informal settlements, rural areas in KwaZulu-Natal are characterized by poor infrastructure and minimal or lack of basic services such as water, sanitation, health and education (Mtshali, 2002). According to recent statistics, KwaZulu-Natal is estimated to have a rural population of 52.5% (Trade and Investment KwaZulu-Natal, 2020).

### Study participants

The study participants were young men aged 18–30. South Africa defines individuals between 15–35 as youths and this age group to be disproportionately affected by high unemployment and generally limited opportunities (Stats SA, 2021). The inclusion criteria for study participants were aged 18–30, male, normally resident in their communities and able to communicate in English and/or isiZulu. The exclusion criteria for study participants were the inability or unwillingness to provide informed consent and lying outside the 18–30 years age range.

### Sampling methods

Snowball sampling was employed to recruit participants. In both sites, initial contact was made through Project Empower, a local non-governmental organisation working with young men and women on issues including gender-based violence and HIV prevention. Project Empower supported identification of initial participants (‘seeds’) who met the study criteria. These initial participants then referred peers from their neighbourhoods and social networks, allowing recruitment to extend beyond individuals directly connected to Project Empower.

Snowballing was particularly appropriate given the absence of a clear sampling frame and the hard-to-reach nature of some community segments (Naderifar et al., 2017).

### Data collection and data analysis

We conducted 22 interviews (10 rural and 12 urban informal settlements) between June and July 2022. Interviews were conducted at the Project Empower premises in an urban site and at a centrally hired hall in a rural site. The interviews were conducted in isiZulu by a trained male interviewer (SM) using a semi-structured interview guide. In this context, young men often suppress emotional vulnerability, but when they do share personal or sensitive experiences such as their emotional struggles and sexual behaviour, they are culturally more likely to do so with another man. Masculinity norms in KwaZulu-Natal can emphasise emotional restraint and strength in front of women, and participants may have perceived female interviewers as less able to ‘handle’ distressing information or may have felt pressure to maintain a stoic identity. Additionally, the interviewer had extensive experience in conducting sensitive interviews, facilitating rapport and encouraging open discussion with young men. The semi-structured interview guide asked young men about their reasons for being upset and how they regulate their emotions during this state, guided by the six domains of ED in the DERS-16: non-acceptance of negative emotions, inability to engage in goal-directed behaviours when distressed, difficulties controlling impulsive behaviours when distressed, limited access to emotion regulation strategies perceived as effective, lack of emotional awareness, and lack of emotional clarity. The interviews were audio recorded, and the interviewer took notes on emphasized phrases during the interviews.

### Data analysis

We did descriptive analysis for the demographic data and thematic analysis for the qualitative data. The interviews were transcribed verbatim, and the isiZulu transcripts were translated into English by a professional transcriber for analysis. To understand how men regulate their emotions, we used deductive thematic analysis using the six main domains of ED in the DERS-16 as the main themes, identifying codes within the main themes, and then clustering these codes into sub-themes related to the main themes. In addition to these predefined domains, an emerging theme on the lack of mental health services for men was identified inductively during the analysis. This theme was retained to capture important contextual insights that extended beyond the DERS-16 framework. All data analysis was conducted manually.

### Trustworthiness and reflexivity

Four criteria of trustworthiness including credibility, dependability, confirmability and transferability were employed to ensure study rigor. Credibility was enhanced by obtaining rich, detailed descriptions of how men regulate their emotions, triangulating data from diverse sources (i.e. men from rural and urban informal settlements), employing member checking during data collection, and engaging in peer debriefing throughout data analysis. Dependability was ensured through the consistent use of a semi-structured interview guide and thorough documentation of the research process, from data collection to analysis. Confirmability was maintained by audio-recording interviews, taking detailed notes, and transcribing verbatim to accurately reflect participants’ perspectives and support findings with direct quotations. Finally, transferability was addressed through thick description of the data, contextualizing findings by clearly linking them to existing literature and previous studies, thus enabling comparisons with other contexts or populations.

Reflexivity was incorporated by acknowledging how researcher identity and cultural positioning may influence data generation and interpretation. The male interviewer’s familiarity with local norms and language supported rapport and comfort discussing sensitive emotional topics, while ongoing reflexive discussion helped the team remain aware of how gender and positionality could shape participant responses and analytic decisions.

### Ethics

The study was approved by South Africa Medical Research Council (EC023-10/2022) and University of KwaZulu-Natal Biomedical Research Ethics Committee (BREC/00004912/2022). All participants provided written informed consent prior to participation and any information such as their real names which can help identify them were not used during the study. In line with our ethical approval and informed consent procedures, we had protocols in place to manage any sensitive disclosures that could arise during interviews. Participants were informed that if they disclosed distress, safety concerns, or experiences requiring support such as IPV, they would be offered information and referral to appropriate counselling or support services.

## Results

A total of 22 interviews were conducted, 10 in the rural community and 12 in the urban site. Most of the participants (81.82%) were in the 21–24 years range. All participants, except 1, were in a relationship that they identified as heterosexual, and 50% reported that they had children. Just above half of the participants (54.55%) had completed matric. None of the participants reported being formally employed at the time of the study (Table 1).

### Overview of the qualitative findings

Men’s adversity was rooted largely in their past hardships, particularly adverse childhood experiences and in current structural limitations, especially the lack of opportunities to work and provide. Importantly, situations that left them feeling upset were not limited to immediate triggers, but connected to deeper concerns about identity, responsibility, and belonging. Being ‘upset’ often reflected frustration, shame, and distress linked to their ability to perform expected masculine roles, particularly as providers and protectors. Thus, emotional upset was closely tied to both past experiences and ongoing structural constraints, shaping how men understood and managed their emotions.

Early-life challenges, particularly father absence and limited familial support were frequently cited as sources of ongoing distress. For many, these experiences represented not only emotional loss but a pathway to current disadvantage and restricted life opportunities:

Respondent 2(urban): Yes, let me make an example. Maybe if my dad was present in my life, my life would not have turned out like this. I only have my mother, and she is unemployed. But if maybe Dad was around, he was going to provide for us. A man is the one who is supposed to provide and support his family. If Dad was around, maybe some of the things in my life would have turned out fine like going to varsity [University] and getting employed.

Here, adversity is understood as intergenerational disruption and loss of guidance, linked by participants to their current struggles and feelings of being ‘left behind’. Alongside childhood experiences, men emphasised present-day financial strain, unemployment and inability to provide as central to their emotional distress. These challenges were not only practical concerns but also deeply tied to masculine identity and social status:

Respondent 5(urban): Not having money makes me angry and I get upset. I end up feeling weak. A father of two with no money.

Another participant echoed the emotional burden of failing to meet provider expectations:

Respondent 2(rural): I get angry and stressed because I’m unemployed and have a child. I wonder how I will raise my child. The mother might be working, and she is raising the child, which does not sit well with me. I want to be the one that provides.

Across accounts, adversity was characterised by economic exclusion, disrupted pathways to adulthood and compromised ability to enact expected masculine roles, all of which triggered emotional strain and informed men’s regulation strategies.

In the context of being upset, men described often struggling to understand their emotions (e.g. experiencing multiple emotions at once and failing to differentiate the emotions) which led them to being overwhelmed with emotion linked to lack of emotional clarity, awareness and non-acceptance. These patterns reflected difficulties with emotional clarity, awareness, and acceptance, which align with core DERS-16 domains. Young men also showed that they lacked effective strategies for emotion regulation, another ED dimension of DERS-16, and most of the strategies they used (e.g. numbing with alcohol or sex, and suppressing emotions) reflected dominant masculine norms in this context, where emotional restraint, self-reliance, and the avoidance of vulnerability are culturally valued. These expectations made suppression and numbing socially acceptable ways for men to cope, even though they often aligned with maladaptive regulation patterns described in the DERS-16. The inability to regulate their emotions effectively presented challenges for men; men tended to struggle with impulsivity and often reported perpetrating violence against a range of people (often female partners). Moreover, their ineffectively regulated emotions had a potential of inhibiting the men from achieving some of their goals for instance damaging their crucial relationships and also led to men *s*truggling to engage in goal-orientated ways, which threatened their potential to establish stronger livelihoods reflecting DERS-16 dimensions related to impulse control and difficulty engaging in goal-oriented action. We describe the men’s emotion regulation strategies in-depth below using the domains of ED in the DERS-16 as the main themes.

### Theme 1: lack of emotional clarity and awareness, and non-acceptance of negative emotions

Men often struggled to recognise and differentiate emotional states when upset, particularly anger and related forms of distress. Emotional experiences were frequently described in broad and overlapping terms, which contributed to confusion, difficulty identifying causes of distress, and challenges regulating emotions.

Across interviews, men tended to collapse different emotional states into a single category, often using *ukudinwa* and *ukucasuka* interchangeably. Although these isiZulu terms roughly translate to ‘anger’ and ‘being upset’ respectively, they carry layered meanings related to irritation, emotional discomfort, and exhaustion. This suggests that emotional language may be limited not because emotions are absent, but because culturally embedded concepts do not map neatly onto English emotion labels. As one participant explained:

Interviewer: Would you say that being angry [ukudinwa] and being upset [ukucasuka] is the same thing?

Respondent 3(urban): I would say they are the same.

For some men, emotional confusion stemmed from being upset without fully understanding the source. Rather than a lack of emotion, this reflected difficulty in emotional clarity:

Interviewer: Alright, I hear you. When you are by yourself, do you ever get upset? Respondent 6 (rural): Yes

Interviewer: What causes that?

Respondent 6(rural): I’m not sure what the cause might be, I try to figure out the cause.

In some cases, men were unable to define emotional terms entirely:

Interviewer: Hmm, ay alright. So, do you know the term ‘anger’ [ukudinwa] what it means?

Respondent 1(urban): [silence] No, I don’t know what it means.

Mixed emotional states were also common. Men described feeling anger and upset simultaneously, which was overwhelming and associated with loss of control, linking directly to emotional clarity and impulse control difficulties:

Interviewer: Do you ever feel ukucasuka [upset] and nokudinwa [angry] all at once?

Respondent 3(rural): Yes. When I feel like that, it becomes very dangerous, to the point that I may end up making a bad decision, even leading to an altercation. It’s like putting petrol next to a fire – you cannot expect there won’t be an explosion. It’s better to pair fire with water, as those two elements complement each other instead of working against one another. So, anger [ukudinwa] combined with annoyance [ukucasuka] is truly dangerous. I’ve experienced these emotions before, and found myself losing control, acting impulsively, and making decisions that led to negative outcomes.

Some men had difficulty accepting negative emotions. While this was not explicitly discussed by men, they showed it through worrying about, and constantly questioning, why they were feeling emotional:

Respondent 7(urban): You tend to ask yourself, why am I feeling upset? It makes me feel worried.

Beyond confusion, men also showed difficulty accepting negative emotions. Rather than viewing feeling upset as a natural human experience, participants described it as abnormal or unacceptable:

Interviewer: Ok. Do you think that being upset is a normal thing?

Respondent 4 (urban): Ah, it is not.

Rejecting negative emotions appeared tied to masculine expectations that men should remain composed, sociable, and unaffected by hardship. Participants worried about why they felt emotional, questioning themselves and attempting to suppress distress:

Interviewer: So, do you think that being upset is normal?

Respondent 2(urban): No, it’s not a normal feeling. That thing is not normal.

Interviewer: Okay, and why do you say that it is not a normal feeling?

Respondent 2(urban): Because when you’re feeling that way, you can’t feel yourself. It’s not something that I would want to feel. If it were up to me, I would always be happy, laughing and socializing with people.

Overall, these accounts illustrate how limited emotional vocabulary, overlapping cultural meanings of emotion terminology and discomfort with acknowledging negative feelings contribute to difficulties in emotional clarity, awareness, and acceptance. Rather than an absence of emotion, men’s descriptions reflect a constrained ability to label and interpret emotional states. These culturally shaped patterns of expression may hinder men’s ability to identify triggers, understand their internal states, and regulate emotions effectively, increasing vulnerability to impulsive or maladaptive responses.

### Theme 2: lack of confidence in, or limited access to effective emotion regulation skills

Men described a range of strategies for managing emotions when upset; however, these were often shaped by dominant constructions of masculinity that prioritise emotional control, stoicism, and self-reliance. As a result, many relied on suppression and avoidance, strategies that may offer short-term relief but hinder emotional processing and heighten vulnerability to ED.

Suppressing emotions, particularly not crying or not showing visible distress, was widely regarded as expected masculine behaviour. Crying was seen as incompatible with strength and maturity, and men described feeling pressure to handle emotional difficulties independently. These expectations appeared to shape emotional responses and contributed to internalised beliefs that expressing vulnerability was unacceptable:

Interviewer: Why don’t you believe in crying?

Respondent 2(urban): It’s just that I don’t believe a man should cry – there’s simply no reason for it. Although you might sometimes feel the urge to cry, as a man, you cannot allow yourself to do so. Even in your mind, you understand that crying isn’t for men; it’s something associated with women. If you’re a man, you’re expected to face your problems directly; crying serves no purpose. You reach a point where you’re unsure how to feel because your mind becomes numb, yet crying is still not an acceptable option for a man.

Similarly, others emphasised privacy and emotional self-containment, expressing discomfort with appearing vulnerable or being pitied:

Respondent 7(rural): I keep my business private. I do not share my personal things with people. If there is a specific situation I want to get their advice on, I just pretend I am asking for someone else, and they would tell me ‘hypothetically’ what they might do in that situation. I don’t like it when someone pities me which may even make me emotional. I do not like that at all. As a man, I like being self-reliant.

Alongside suppression, men also described using alcohol, cannabis, and sex to numb or escape feelings of being upset. These behaviours appeared socially acceptable within peer groups and offered temporary emotional relief, though participants recognised their potential harm. In several cases, men shifted between multiple avoidance-based strategies, illustrating attempts to cope without tools for emotional processing:

Interviewer: When speaking about strategies to control your emotions, what do you do to make yourself feel better?

Respondent 12(urban): Besides seeing friends and smoking weed?

Interviewer: Yes

Respondent 12(urban): Let me be specific. When I am angry, I have sex. I call my girlfriend and ask her where she is. After sex everything subsides.

Interviewer: Besides that, is there anything else?

Respondent 12(urban): There is, but I realised that it is bad. I cannot get drunk every time I am angry.

Interviewer: So, let us say you are not in good books with your girlfriend, what do you do to feel better?

Respondent 12(urban): Ok, as a young guy, I am still playing games and do not have a person I am committed to. I just go to another person immediately and enjoy myself.

Another participant described how he used alcohol to escape his negative emotions and how it soothed his mind:

Interviewer: Alright, let us proceed, what are the things that you often turn to make yourself feel better when you are angry?

Respondent 10 (rural): It is better to turn to alcohol most of the times, it eases the mind [Ukuhleka].

While most men relied on avoidance-based strategies, a few men described adaptive strategies such as music and sport. These alternative coping practices were less frequent, often limited by context, and sometimes emerged as individual improvisations rather than learned emotional skills:

Respondent 1(rural): What I do is I play music, or I go and play soccer and be around friends.

Taken together, these narratives suggest that when upset, men often lacked confidence in adaptive emotional coping strategies and relied on culturally reinforced responses centred on self-control, concealment, and avoidance. Although these approaches align with masculine expectations of strength and self-reliance, they limited opportunities for emotional reflection and left men with few accessible tools when distressed, likely leading to the development of ED over time.

### Theme 3: difficulties controlling impulsive behaviours when distressed

A common pattern in the interviews was a limited capacity to control impulses when upset, with men describing acts carried out in the heat of the moment that they later regretted. These behaviours ranged from verbal aggression and destruction of property to physical violence against intimate partners and fights with other men. This cluster of responses illustrates how heightened emotional arousal, limited skills for calming, and social expectations about masculine responses combined to produce risky actions.

Men described different types of impulsive acts with different antecedents and consequences. One form was partner-directed physical violence often triggered by jealousy, suspicion, or perceived disrespect. Several participants framed such violence as an immediate, unplanned reaction during intense upset, sometimes followed by remorse when the facts became clear:

Respondent 8(urban): When I am upset, I reflect deeply on what’s troubling me and how it makes me feel, then I try to address the issue directly to avoid repeating the mistake. For example, I once hit my girlfriend because I suspected her of cheating. When I returned home, she explained the truth that the person who had called her was actually her brother but in my state of anger, I didn’t trust her words and reacted violently. Later, once the anger subsided, I came to my senses and realised it genuinely was her brother”.

Here, the antecedent is a perceived threat to status or relationship trust; the mechanism being intense arousal combined with an impulse to reassert control leading to harming of the relationship and later regret. This pattern highlights how difficulty processing emotions can escalate into impulsive acts.

Another cluster of behaviours involved interpersonal fights with other men, sometimes with severe consequences. These incidents were frequently described as fast-moving confrontations in which escalation was swift and de-escalation skills were limited:

Respondent 7 (urban): To be honest, the one who came home ended up being stabbed by my brother, so we could have ended up killing him. He apologized and that passed. But during that time, we could have killed him.

Such episodes point to the intersection of anger, group dynamics, and sometimes weapon use, demonstrating a different risk profile and severity compared with other impulsive acts.

A third pattern was workplace or public verbal aggression, rude or aggressive speech that could jeopardise employment or social standing. Participants portrayed these acts as momentary losses of control, often provoked by repeated irritation or perceived disrespect:

Interviewer: So, what do you do in that situation when you’re angry at work?

Respondent 12(urban): I confront the person who has made me angry, and I speak to them in a rude tone. Initially, they might instruct me politely, and I comply, thinking it won’t happen again. But when I return the next day and they continue doing the same thing, that’s when I become angry. At that point, my tone becomes very aggressive. When I’m angry, I don’t care about age or anything else – I simply say whatever I feel like saying.

Finally, some men channelled distress into destruction of objects or withdrawal to sleep, behaviours that are less directly harmful to others but still signal dysregulated coping:

Responded 6(rural): … when I get angry, I just want to go to bed and just sleep it off and if I can’t sleep, I get up and find something to destroy’.

Across accounts, the triggers for impulsive reactions varied, including jealousy, perceived betrayal, ongoing tension in relationships, accumulated provocation, and substance use. This indicates that impulsive acts were not uniform, but influenced by different emotional, relational, and situational factors. Some men expressed remorse afterwards or described attempting to repair relationships once calm, suggesting emotional reflection and awareness following loss of control. Masculine expectations around defending one’s dignity and appearing strong in social situations also shaped these reactions, making forceful responses feel necessary or justified in the moment. At the same time, alcohol and cannabis sometimes intensified emotional arousal, reducing the ability to pause and increasing the likelihood of acting quickly when upset.

Taken together, these narratives illustrate how emotional intensity, social expectations, and limited calming strategies contributed to rapid and sometimes harmful responses when distressed. While participants often regretted these actions afterwards, they described difficulty interrupting emotional escalation in the moment, indicating challenges with impulse control under heightened emotional states.

### Theme 4: difficulty engaging in goal-oriented behaviours

Men described how feeling upset interfered with their ability to stay focused on goals and to make decisions that supported their long-term aspirations. When distressed, participants often acted in ways that conflicted with what they valued such as maintaining relationships, staying employed, or progressing educationally. Loss of control in moments of emotional intensity disrupted important personal and interpersonal milestones, sometimes with lasting consequences.

Men often recognised that impulsive actions during emotional distress undermined romantic and family relationships they deeply valued. Several described intimate relationships ending after violent incidents, and a sense of regret emerging once emotions subsided:

Respondent 2(rural): You have a girl that you truly love, and they teach you things. She ended up leaving me because I hit her. I ended up building a wall and developing negative emotions towards other girls. I ended up comparing those girls to this one. The way in which we broke up really upset me.

Likewise, another man also described how he felt so remorseful after being violent towards his girlfriend, but only much later after she had left him:

Respondent 12(urban): For example, I saw a text on my girlfriend’s phone. Instead of asking her what was going on, I just hit her. I later realised that I overreacted and the person I hit was no longer next to me. I felt like I was supposed to ignore her, I had not truly decided what I wish to do, I was reactive. I regretted, imagine she was the mother of my child and I only realised my mistake after two years.

Some men also described how their violence towards others negatively impacted their families. For instance, one of the participants described how he lost one of his family members because of a previous fight:

Respondent 5(urban): Yes. I once hit someone, and his family intervened. I thought things were over, but he shot my other family member and died. I did not know why, and people told me that it is because he fought with me.

One man also described how there was a threat of being arrested and put into jail because of his violence, which could have impacted his future life:

Respondent 12(urban): What made me not go to prison is because I was underage. We were in a holding cell for three days. We were drunk and we came across some guy who was rude towards us. We fought with him and I ended up stabbing him.

In addition to relational and legal consequences, men described struggling to perform tasks when upset, which affected work and schooling. For some, emotional distress led to refusal to comply at work or abrupt decisions that jeopardised employment:

Interviewer: OK. So, let us talk about you being upset [ucasukile] and you are at work, are you able to complete tasks?

Respondent 5(urban): I cannot. My boss once found me sitting down, and my colleagues advised me not to react negatively, suggesting I should practice tolerance. But I just dismissed them and said I won’t listen – they’re not my boss. Even if it were my boss addressing me, I’d simply tell him straight that I’m quitting.

Another man explained how emotional preoccupation interfered with concentration, leading to mistakes and poor performances:

Interviewer: Alright, when you find yourself angry at workplace, can concentrate at work?

Respondent 10(rural): No, I cannot concentrate especially when I am angry because instead of focusing in doing my job, there are things that comes up to mind that makes me lose concentration and I end up making mistakes that could have been avoided.

While most of the men failed to complete their matric (high school), a few were proceeding with their education beyond the matric, and some of the men described experiencing difficulties in concentrating with their studies when upset. For instance, respondent 1 (rural) described this: ‘*When you are angry you can no longer study, how can one study while they are angry, I really cannot even if I try the thing, what I am angry about keeps coming back’*.

Across accounts, emotional intensity disrupted planning, reduced the ability to stay focused on meaningful tasks, and sometimes prompted decisions that worked against men’s own aspirations. While men often recognised the longer-term costs afterwards, they struggled to pause or re-direct themselves in the moment, illustrating challenges sustaining goal-directed behaviour when distressed. These reflections suggest that while men often held clear aspirations, emotional distress disrupted their ability to act in ways aligned with those goals, reinforcing cycles of frustration, loss, and self-doubt.

### Additional theme: limited access to emotional support and mental health services

Although the analysis was guided by the DERS-16 domains, an additional theme emerged across interviews that did not map directly onto the framework: men described having very limited access to emotional support and formal mental health services. This lack of support shaped how men experienced and managed emotional distress, often leaving them without guidance or tools for coping when upset. For some participants, the interview itself appeared to be one of the few opportunities to express emotions openly and be listened to.

One participant directly asked the interviewer for strategies to help him manage his anger, highlighting both a willingness to seek support and the absence of available services:

Respondent 6(rural): I would like to know what sort of things I can do to control my anger

Another participant described the interview as a rare space where he felt able to talk about issues he had been carrying alone:

Respondent 3(urban): I understand what we are doing and it makes me happy because I can speak about things I have been holding in. I feel good and I understood everything.

These accounts suggest that, beyond individual emotion regulation challenges, structural factors such as the absence of accessible psychosocial support and ability to build meaningful friendships contribute to men’s difficulties regulating emotions when upset. The emergence of this theme underscores how emotional experiences are shaped not only by individual strategies, but also by broader contextual limitations in rural and urban informal communities.

## Discussion

This study sought to understand emotion regulation strategies among young men in rural areas and urban informal settlements in KwaZulu-Natal by drawing on the domains of the DERS-16. Overall, the framework aligned well with men’s lived experiences, although some culturally shaped expressions and regulatory practices expanded or nuanced the DERS-16 constructs in ways not captured by the original scale.

Most men reported that their upset and anger stemmed from adverse childhood experiences and poverty, struggling to regulate their emotions during these times. They faced multiple challenges growing up, such as the absence of a father, which reflects previous research on the negative life events young men in South Africa encounter (Mbobo, 2022; Mkhwanazi et al., 2024). Many believed that their childhood hardships contributed to their current issues, like unemployment and inability to provide, further fuelling their anger. Previous work has similarly identified how young men described how their experiences of growing up without a father impacted their current situation, that is, being economically marginalised among other challenges (Mkhwanazi et al., 2024). Adverse childhood experiences are an established risk factor for poor mental health, including ED (Otwell-Dove, 2019) and have also been associated with poverty later in life (Liao et al., 2021) which in turn increases the risk of developing ED (Herts et al., 2012).

Throughout the interviews, it was evident that young men’s emotion regulation strategies, such as suppressing emotions and numbing them with alcohol, substances, and sex, were closely tied to their construction of masculinity (Gibbs et al., 2014). Men described suppressing emotions, particularly in front of others, reflecting a belief that they should be brave and stoic in adversity (Gibbs et al., 2022). Men also employed avoidant coping strategies, such as numbing emotions with alcohol, substances, and sex, which reflected dominant masculinities and prioritized these as ‘safe’ approaches to complex emotions (Gibbs et al., 2014). Emotional restraint, self-reliance, and the avoidance of outward vulnerability are culturally valued within isiZulu and broader African masculine norms (Ezeugwu & Ojedokun, 2020; Moonsamy & Thavanesi, 2024). These expectations help explain why suppression and avoidance were widely adopted and seen as appropriate responses, even though they often align with DERS-16 domains linked to poor regulation such as non-acceptance of emotions and limited access to effective emotion regulation strategies. While suppressing emotions and avoidance coping may be important for young men in challenging contexts, they are understood as unhealthy strategies for regulating emotions, often worsening an individual’s mental health state and well-being (Butler et al., 2007; Neilson et al., 2023). Moreover, given the high rates of HIV infection in South Africa, the use of sex and substances may increase the likelihood of young men acquiring HIV (Browne & Wechsberg, 2010). It is important to note, however, that masculinity in this setting was not experienced purely as harmful; for some men, ideals such as endurance, responsibility, and relational loyalty also supported positive coping strategies, including physical activity, socialising with trusted peers, and listening to music – approaches that reinforced belonging and emotional recovery. Physical activity, peer influence and listening to music have been reported to enhance positive emotion regulation and improve well-being (Carvalho et al., 2022; Sahi et al., 2023; Wu et al., 2022; Zhang et al., 2019).

Another central concept that emerged from the data was the role of violence as a form of impulsive behaviour, reflecting another key construct of ED. In this setting, IPV perpetration is high (Gibbs et al., 2020), and considered a relatively acceptable way of enforcing authority and maintaining power over women. While the majority of research has focused on describing how men’s masculinities are intimately tied to IPV perpetration (Jewkes & Morrell, 2018), this study suggests that high rates of IPV may partly be explained by men’s ED. Quantitative studies in other settings have found an association between IPV perpetration and ED (Maloney et al., 2023; Neilson et al., 2023). Other studies have also reported that general violent behaviour is associated with ED (Garofalo et al., 2021). Identifying whether ED is associated with IPV remains an important issue in this population.

Throughout the interviews, men often described how their impulsive behaviours or unresolved and overwhelming emotions often inhibited them from achieving some of their goals which is linked to DERS-16’s ED dimension on inability to engage in goal-oriented behaviours. For instance, men damaged relationships that they valued, put the lives of their families in danger, and risked being arrested because of their violent behaviour. Likewise, other studies have highlighted how impulsive behaviours can negatively affect interpersonal relationships (Aan Het Rot et al., 2015) and place oneself and others at risk of harm (Chamorro et al., 2012). In addition, men described how their inability to concentrate and finish work and school-related tasks impacted their work and educational goals, potentially inhibiting ways in which men could establish stronger livelihoods. Other studies have also identified impulsivity as negatively associated with academic performance (Lozano et al., 2014) and work performance (Schweizer, 2002).

An additional theme that sat slightly outside the DERS-16 framework was young men’s limited access to emotional support and mental health services. Several participants expressed a desire for guidance on how to manage anger, suggesting that formal and informal avenues for emotional support were scarce. This aligns with broader evidence showing that access to mental health services in South Africa remains severely constrained, particularly for young individuals in rural and low-resource settings (Chidi, 2025). Limited service availability often intersects with gendered stigma around help-seeking, where norms of self-reliance and emotional restraint discourage men from seeking professional support (Mokhwelepa & Sumbane, 2025). This contextual barrier likely reinforces reliance on suppressive or avoidant coping strategies and restricts opportunities to develop adaptive regulation skills. The lack of psychosocial support also highlights a contextual dimension of ED not fully captured by the DERS-16, which primarily focuses on individual-level processes.

The DERS-16 offered a useful analytic structure; overall, this study highlights that emotional experiences in this context mapped onto several DERS-16 domains. Some domains, particularly those related to limited access to effective emotion regulation strategies, impulse control, and goal-directed behaviour aligned closely with men’s lived experiences. However, other aspects of emotional expression, such as the fluid, overlapping meanings of *ukudinwa* (anger/exhaustion) and *ukucasuka* (annoyance/upset), extended beyond the scale’s original framing. These nuances suggest that while the DERS-16 captures broad patterns of ED in this context, certain culturally embedded expressions and coping practices are not fully reflected in the instrument. This highlights the importance of contextual interpretation when applying Western-developed frameworks in African settings (Kohrt et al., 2014).

Researcher positionality was central to the interpretation of the findings. Interviews were conducted by a male, isiZulu-speaking researcher with extensive experience working with young men in similar communities. His shared sex, language, and cultural background helped establish rapport and enabled participants to express emotions using idioms and concepts that may not translate directly into English, a process recognised in qualitative research as enhancing cultural congruence and depth of disclosure (Redman MacLaren et al., 2014). In addition to the interviewer, the wider research team comprised three members with different cultural backgrounds, sexes, disciplinary positions, and who did not speak isiZulu. This diversity created both distance and complementarity, offering alternative readings of the data while also requiring continuous reflexive engagement to avoid imposing outsider interpretations. The team reflected on how their respective identities, lack of linguistic fluency, and experiences working in similar (or different) contexts shaped coding decisions, the interpretation of culturally specific emotion terms (such as ukudinwa and ukucasuka) and the framing of men’s emotional experiences. Maintaining reflexive awareness throughout analysis allowed the team to consider how positionality influences meaning-making and interpretive choices, consistent with recommendations for rigorous qualitative inquiry (Braund et al., 2024). This reflexive stance informed both the analytic process and the framing of emotional experiences within the DERS-16 domains.

### Study limitations

Our study had several limitations. First, it used a non-representative and limited sample, which makes it difficult to generalize the findings to all young men in South Africa. However, this was not the aim of qualitative research; rather, the nature of the interviews allowed us to gain a deep understanding of how a small group of young men experienced ED and the strategies they used to regulate their emotions. Second, the use of a deductive analytic approach based on the DERS-16 framework may have constrained the identification of culturally specific or novel expressions of ED. However, because our analytic process retained some flexibility, we were still able to identify an additional emerging theme which fell outside the DERS-16 domains but was important in understanding men’s broader emotion-regulation context. Finally, while the inclusion of an isiZulu-speaking male interviewer enhanced rapport and depth of responses, shared cultural and gendered positionalities may also have influenced the type and framing of disclosures, particularly around sensitive issues such as masculinity and emotional vulnerability. Reflexive measures were used to mitigate this, but researcher positionality remains an important consideration in interpreting the findings.

### Study implications

Despite these limitations, our study has several important implications. Firstly, young men living in marginalised communities in South Africa appear to struggle with ED. Secondly, our findings point to how adverse childhood experiences, poverty, and constructions of masculinity act as structural drivers of poor emotion regulation among young men in resource-limited settings. Thirdly, the study underscores the detrimental effects of ED on men’s mental health and its potential contribution to IPV and HIV risk. Fourth, the findings also reveal a lack of accessible and gender-responsive mental health services tailored to men’s needs in these contexts. Many participants expressed difficulty accessing emotional or psychological support, highlighting the need to integrate mental health services within community and primary health systems in ways that resonate with men’s lived experiences. Lastly, the framework of the DERS-16 conceptually mapped how young men experienced ED in this context and, as such, may be an appropriate tool to measure and understand ED in this population.

## Conclusion

Men who grew up and live in resource-constrained environments might be struggling to regulate their negative emotions in a healthy way, with adverse childhood experiences and the contexts of poverty associated with living in such areas having a major influence on their emotion regulation capabilities. Some men’s masculinities, linked to emotional suppression and avoidance coping in men, also seem to shape emotion regulation. Poor emotion regulation can have a detrimental effect on men’s mental health and overall well-being. Additionally, ED among men results in acts of violence, including against their intimate partners, and can increase their risk of HIV acquisition, which is particularly concerning, as both IPV and HIV are considerable problems in SA, especially in men’s contexts. These preliminary data suggest that ED among men in resource-constrained contexts merits further investigation.

## Figures and Tables

**Table 1:** Demographic characteristics of participants (*N* = 22)

Socio-demographics	n (%)
Age	
21-24 years	18(81.82)
≥25 years	4(18.18)
Education level	
Primary & Secondary	6(27.27)
Matric	12(54.55)
College	4(18.18)
Residence	
Urban	12(54.55)
Rural	10(45.45)
Relationship	
In a relationship	21(95.45)
Single	1(4.55)
Number of children	
0	11(0.50)
1	8(36.36)
2	3(13.64)
